# *catena*-Poly[cadmium-μ-[1,3-bis­(imidazol-1-yl)propane]-di-μ-chlorido]

**DOI:** 10.1107/S1600536812021083

**Published:** 2012-05-16

**Authors:** Qiao-Lian Shen, Hong Lin

**Affiliations:** a Jinhua Radio and Television University, Jinhua, Zhejiang 321022, People’s Republic of China; b Jinhua Professional–Technical College, Jinhua, Zhejiang 321007, People’s Republic of China

## Abstract

The title complex, [CdCl_2_(C_9_H_12_N_4_)]_*n*_, is characterized by the formation of a zigzag chain structure parallel to [001]. In the chain, the Cd^2+^ cation is coordinated by four bridging Cl^−^ ligands in equatorial positions and two N atoms from symmetry-related and likewise bridging 1,3-bis­(imidazol-1-yl)propane ligands in axial positions, forming a distorted CdCl_4_N_2_ octa­hedron.

## Related literature

For related structures, see: Carlucci *et al.* (1997 ▶); Wang *et al.* (2011 ▶); Yang *et al.* (2010 ▶).
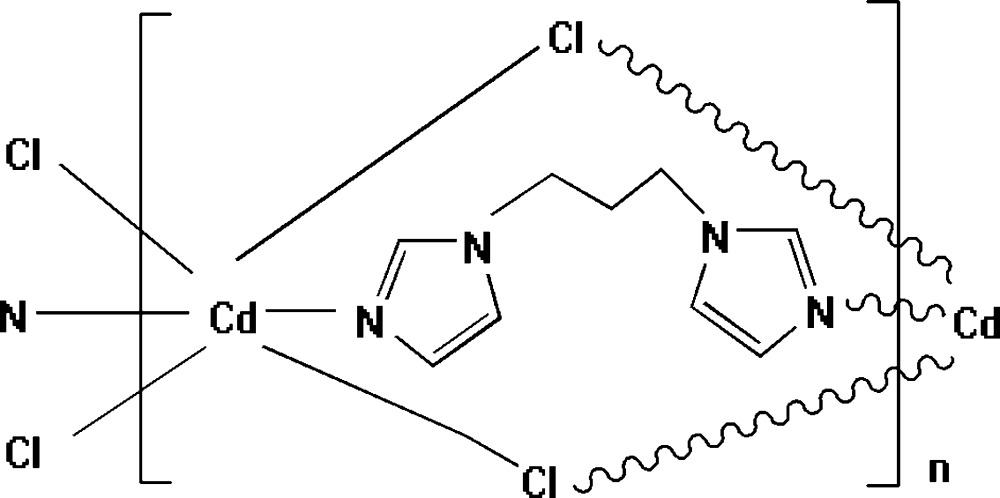


## Experimental

### 

#### Crystal data

[CdCl_2_(C_9_H_12_N_4_)]
*M* *_r_* = 359.54
Orthorhombic, 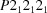

*a* = 15.1617 (16) Å
*b* = 9.9810 (11) Å
*c* = 7.8022 (8) Å
*V* = 1180.7 (2) Å^3^
*Z* = 4
Mo *K*α radiation
μ = 2.28 mm^−1^
*T* = 296 K
0.31 × 0.20 × 0.12 mm

#### Data collection

Bruker APEXII area-detector diffractometer
Absorption correction: multi-scan (*SADABS*; Sheldrick, 1996 ▶) *T*_min_ = 0.585, *T*_max_ = 0.761
18692 measured reflections
2707 independent reflections
2645 reflections with *I* > 2σ(*I*)
*R*_int_ = 0.021

#### Refinement

*R*[*F* ^2^ > 2σ(*F* ^2^)] = 0.013
*wR*(*F* ^2^) = 0.037
*S* = 1.00
2707 reflections
146 parameters
H-atom parameters constrained
Δρ_max_ = 0.20 e Å^−3^
Δρ_min_ = −0.47 e Å^−3^
Absolute structure: Flack (1983 ▶), 1109 Friedel pairs
Flack parameter: 0.294 (18)

### 

Data collection: *APEX2* (Bruker, 2006 ▶); cell refinement: *SAINT* (Bruker, 2006 ▶); data reduction: *SAINT*; program(s) used to solve structure: *SHELXS97* (Sheldrick, 2008 ▶); program(s) used to refine structure: *SHELXL97* (Sheldrick, 2008 ▶); molecular graphics: *DIAMOND* (Brandenburg, 2006 ▶); software used to prepare material for publication: *SHELXTL* (Sheldrick, 2008 ▶).

## Supplementary Material

Crystal structure: contains datablock(s) I, global. DOI: 10.1107/S1600536812021083/wm2624sup1.cif

Structure factors: contains datablock(s) I. DOI: 10.1107/S1600536812021083/wm2624Isup2.hkl

Additional supplementary materials:  crystallographic information; 3D view; checkCIF report

## Figures and Tables

**Table 1:** Selected bond lengths (Å)

Cd—N2	2.2836 (12)
Cd—N1^i^	2.2911 (13)
Cd—Cl1	2.6370 (4)
Cd—Cl2	2.6800 (4)
Cd—Cl1^ii^	2.7010 (4)
Cd—Cl2^i^	2.7409 (5)

Symmetry codes: (i) 
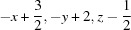
; (ii) 
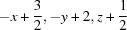
.

**Fig. 1.**
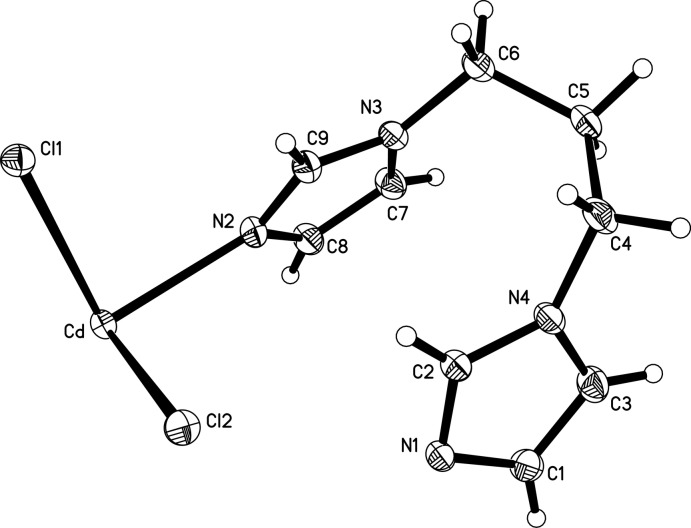
The asymmetric unit of (I), showing the atom-numbering scheme. Displacement ellipsoids are drawn at the 30% probability elevl.

**Fig. 2.**
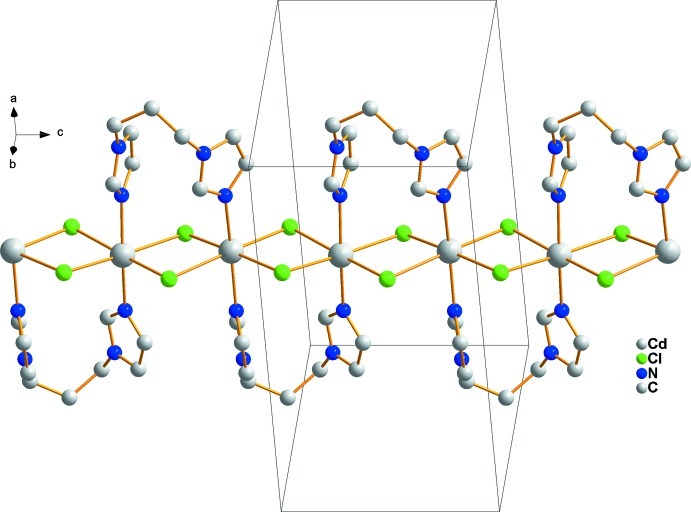
A view of the one-dimensional chain structure of (I).

## supplementary crystallographic information

### Comment

In the past few years, complexes based on the 1,3-bi-4-pyridylpropane (bpp) ligand, such as [Ni_2_(C_10_H_8_O_4_S_2_)_2_(bpp)_2_(H_2_O)]_n_ (Wang *et al.*, 2011), [Ag(C_8_H_9_O_4_)(bpp)]_n_ (Yang *et al.*, 2010), [Ag(bpp)]_n_n(CF_3_SO_3_) (Carlucci *et al.*, 1997), have been reported. However, complexes with 1,3-bis(imidazol-1'-yl)propane (bip) as ligand are scarce. Herein, we report the synthesis and structure of a new complex, [Cd(bip)Cl_2_]_n_ (I).

A perspective view of the molecular entities of compound (I) is presented in Fig.1. The asymmetric unit consists of one Cd^2+^ ion, one 1,3-bis(imidazol-1'-yl)propane ligand, and two chlorine atoms. The Cd^2+^ ion is six-coordinate and has a slightly distorted octahedral coordination environment, defined by four chlorine atoms and two nitrogen atoms from two symmetry-related 1,3-bis(imidazol-1'-yl)propane ligands. As shown in Fig. 2, the adjacent Cd(II) ions are bridged by one 1,3-bis(imidazol-1'-yl)propane ligand and two chlorine atoms to generate a zigzag-chain structure running along [001].

It should be noted that there are no remarkable hydrogen bonding interactions in the crystal.

### Experimental

A mixture of 1,3-bis(imidazol-1'-yl)propane (0.088 g, 0.5 mmol), CdCl_2_^.^2.5H_2_O (0.342 g, 1.5 mmol), and Na_2_CO_3_ (0.060 g, 0.5 mmol) in H_2_O (16 ml)/C_2_H_5_OH (2 ml) was placed in a 25 ml Teflon-lined stainless steel vessel and heated at 433 K for 72 h, then cooled to room temperature over 3 days. Colourless crystals suitable for X-ray analysis were obtained.

### Refinement

The carbon-bound H-atoms were positioned geometrically and included in the refinement using a riding model [aromatic C—H 0.93 Å and aliphatic C—H 0.97 Å, *U*_iso_(H) = 1.2*U*_eq_(C)]. The used intensity data originates from an inversion-twinned crystal.

### Crystal data

**Table:**

[CdCl_2_(C_9_H_12_N_4_)]	*F*(000) = 704
*M**_r_* = 359.54	*D*_x_ = 2.023 Mg m^−^^3^
Orthorhombic, *P*2_1_2_1_2_1_	Mo *K*α radiation, λ = 0.71073 Å
Hall symbol: P 2ac 2ab	Cell parameters from 9933 reflections
*a* = 15.1617 (16) Å	θ = 2.4–27.5°
*b* = 9.9810 (11) Å	µ = 2.28 mm^−^^1^
*c* = 7.8022 (8) Å	*T* = 296 K
*V* = 1180.7 (2) Å^3^	Block, colourless
*Z* = 4	0.31 × 0.20 × 0.12 mm

### Data collection

**Table:**

Bruker APEXII area-detector diffractometer	2707 independent reflections
Radiation source: fine-focus sealed tube	2645 reflections with *I* > 2σ(*I*)
Graphite monochromator	*R*_int_ = 0.021
ω scans	θ_max_ = 27.5°, θ_min_ = 2.4°
Absorption correction: multi-scan (*SADABS*; Sheldrick, 1996)	*h* = −19→19
*T*_min_ = 0.585, *T*_max_ = 0.761	*k* = −12→12
18692 measured reflections	*l* = −10→10

### Refinement

**Table:**

Refinement on *F*^2^	Secondary atom site location: difference Fourier map
Least-squares matrix: full	Hydrogen site location: inferred from neighbouring sites
*R*[*F*^2^ > 2σ(*F*^2^)] = 0.013	H-atom parameters constrained
*wR*(*F*^2^) = 0.037	*w* = 1/[σ^2^(*F*_o_^2^) + (0.024*P*)^2^ + 0.1705*P*] where *P* = (*F*_o_^2^ + 2*F*_c_^2^)/3
*S* = 1.00	(Δ/σ)_max_ = 0.002
2707 reflections	Δρ_max_ = 0.20 e Å^−^^3^
146 parameters	Δρ_min_ = −0.47 e Å^−^^3^
0 restraints	Absolute structure: Flack (1983), 1109 Friedel pairs
Primary atom site location: structure-invariant direct methods	Flack parameter: 0.294 (18)

### Special details

**Table:**

Geometry. All e.s.d.'s (except the e.s.d. in the dihedral angle between two l.s. planes) are estimated using the full covariance matrix. The cell e.s.d.'s are taken into account individually in the estimation of e.s.d.'s in distances, angles and torsion angles; correlations between e.s.d.'s in cell parameters are only used when they are defined by crystal symmetry. An approximate (isotropic) treatment of cell e.s.d.'s is used for estimating e.s.d.'s involving l.s. planes.
Refinement. Refinement of *F*^2^ against ALL reflections. The weighted *R*-factor *wR* and goodness of fit *S* are based on *F*^2^, conventional *R*-factors *R* are based on *F*, with *F* set to zero for negative *F*^2^. The threshold expression of *F*^2^ > σ(*F*^2^) is used only for calculating *R*-factors(gt) *etc*. and is not relevant to the choice of reflections for refinement. *R*-factors based on *F*^2^ are statistically about twice as large as those based on *F*, and *R*- factors based on ALL data will be even larger.

### Fractional atomic coordinates and isotropic or equivalent isotropic displacement parameters (Å^2^)

**Table:**

	*x*	*y*	*z*	*U*_iso_*/*U*_eq_	
Cd	0.743239 (6)	0.998451 (13)	0.383812 (14)	0.02486 (4)	
Cl1	0.77859 (3)	1.17683 (4)	0.14414 (5)	0.02759 (8)	
Cl2	0.76493 (3)	1.18680 (4)	0.62497 (5)	0.02915 (8)	
N1	0.90723 (8)	0.98124 (13)	0.87595 (17)	0.0275 (3)	
N2	0.88887 (8)	0.94055 (13)	0.39339 (19)	0.0275 (3)	
N3	1.03373 (9)	0.95040 (13)	0.40284 (19)	0.0272 (3)	
N4	1.04570 (8)	1.01445 (15)	0.80238 (17)	0.0283 (3)	
C1	0.96006 (11)	0.89687 (17)	0.9691 (2)	0.0325 (4)	
H1B	0.9401	0.8358	1.0504	0.039*	
C2	0.96144 (10)	1.05139 (16)	0.7788 (2)	0.0278 (3)	
H2A	0.9435	1.1182	0.7034	0.033*	
C3	1.04561 (12)	0.91557 (17)	0.9251 (2)	0.0348 (4)	
H3A	1.0944	0.8707	0.9691	0.042*	
C4	1.12411 (11)	1.07572 (18)	0.7238 (2)	0.0360 (4)	
H4A	1.1676	1.0905	0.8129	0.043*	
H4B	1.1076	1.1627	0.6787	0.043*	
C5	1.16718 (9)	0.99572 (18)	0.5806 (2)	0.0339 (3)	
H5B	1.1687	0.9023	0.6148	0.041*	
H5A	1.2278	1.0256	0.5692	0.041*	
C6	1.12330 (9)	1.00460 (18)	0.4051 (2)	0.0325 (3)	
H6A	1.1215	1.0977	0.3696	0.039*	
H6B	1.1589	0.9561	0.3225	0.039*	
C7	1.01061 (11)	0.81997 (16)	0.4383 (2)	0.0321 (3)	
H7A	1.0485	0.7491	0.4622	0.039*	
C8	0.92134 (11)	0.81612 (16)	0.4312 (2)	0.0317 (3)	
H8A	0.8872	0.7401	0.4494	0.038*	
C9	0.95856 (10)	1.01840 (16)	0.3763 (2)	0.0282 (3)	
H9A	0.9560	1.1091	0.3492	0.034*	

### Atomic displacement parameters (Å^2^)

**Table:**

	*U*^11^	*U*^22^	*U*^33^	*U*^12^	*U*^13^	*U*^23^
Cd	0.01757 (6)	0.03157 (7)	0.02543 (7)	0.00113 (4)	−0.00088 (3)	0.00122 (5)
Cl1	0.02733 (19)	0.02735 (16)	0.02807 (19)	−0.00186 (15)	0.00135 (14)	0.00086 (14)
Cl2	0.02980 (16)	0.02852 (17)	0.02914 (19)	−0.00164 (16)	0.00014 (17)	−0.00060 (15)
N1	0.0205 (6)	0.0341 (6)	0.0280 (7)	0.0008 (5)	0.0000 (5)	−0.0041 (7)
N2	0.0214 (6)	0.0324 (6)	0.0287 (7)	0.0032 (5)	−0.0016 (6)	0.0004 (6)
N3	0.0210 (6)	0.0315 (6)	0.0289 (7)	−0.0002 (5)	0.0016 (6)	0.0026 (6)
N4	0.0197 (6)	0.0333 (7)	0.0318 (7)	−0.0011 (6)	0.0018 (5)	−0.0040 (6)
C1	0.0314 (9)	0.0337 (8)	0.0323 (9)	0.0030 (7)	0.0015 (7)	0.0042 (7)
C2	0.0222 (7)	0.0335 (8)	0.0278 (8)	0.0012 (6)	0.0009 (6)	−0.0016 (6)
C3	0.0269 (8)	0.0385 (9)	0.0391 (10)	0.0065 (7)	−0.0032 (7)	0.0002 (8)
C4	0.0226 (8)	0.0414 (9)	0.0439 (10)	−0.0091 (7)	0.0029 (7)	−0.0063 (8)
C5	0.0174 (6)	0.0446 (9)	0.0396 (8)	−0.0008 (7)	0.0019 (6)	−0.0008 (9)
C6	0.0217 (7)	0.0387 (8)	0.0370 (8)	−0.0024 (7)	0.0029 (6)	0.0043 (9)
C7	0.0265 (8)	0.0308 (8)	0.0390 (9)	0.0062 (7)	0.0018 (7)	0.0044 (7)
C8	0.0262 (8)	0.0321 (7)	0.0369 (9)	−0.0003 (7)	0.0009 (6)	0.0025 (7)
C9	0.0235 (7)	0.0312 (7)	0.0301 (8)	0.0025 (6)	−0.0012 (6)	0.0023 (8)

### Geometric parameters (Å, º)

**Table:**

Cd—N2	2.2836 (12)	N4—C4	1.471 (2)
Cd—N1^i^	2.2911 (13)	C1—C3	1.355 (2)
Cd—Cl1	2.6370 (4)	C1—H1B	0.9300
Cd—Cl2	2.6800 (4)	C2—H2A	0.9300
Cd—Cl1^ii^	2.7010 (4)	C3—H3A	0.9300
Cd—Cl2^i^	2.7409 (5)	C4—C5	1.521 (2)
Cl1—Cd^i^	2.7010 (4)	C4—H4A	0.9700
Cl2—Cd^ii^	2.7409 (5)	C4—H4B	0.9700
N1—C2	1.319 (2)	C5—C6	1.525 (2)
N1—C1	1.371 (2)	C5—H5B	0.9700
N1—Cd^ii^	2.2911 (13)	C5—H5A	0.9700
N2—C9	1.318 (2)	C6—H6A	0.9700
N2—C8	1.368 (2)	C6—H6B	0.9700
N3—C9	1.3425 (19)	C7—C8	1.355 (2)
N3—C7	1.376 (2)	C7—H7A	0.9300
N3—C6	1.4618 (19)	C8—H8A	0.9300
N4—C2	1.3423 (19)	C9—H9A	0.9300
N4—C3	1.375 (2)		
			
N2—Cd—N1^i^	170.41 (4)	N1—C2—H2A	124.2
N2—Cd—Cl1	89.86 (4)	N4—C2—H2A	124.2
N1^i^—Cd—Cl1	97.10 (3)	C1—C3—N4	106.03 (14)
N2—Cd—Cl2	92.06 (4)	C1—C3—H3A	127.0
N1^i^—Cd—Cl2	94.53 (3)	N4—C3—H3A	127.0
Cl1—Cd—Cl2	89.962 (15)	N4—C4—C5	115.79 (14)
N2—Cd—Cl1^ii^	85.98 (4)	N4—C4—H4A	108.3
N1^i^—Cd—Cl1^ii^	87.45 (3)	C5—C4—H4A	108.3
Cl1—Cd—Cl1^ii^	174.530 (8)	N4—C4—H4B	108.3
Cl2—Cd—Cl1^ii^	86.642 (15)	C5—C4—H4B	108.3
N2—Cd—Cl2^i^	84.09 (4)	H4A—C4—H4B	107.4
N1^i^—Cd—Cl2^i^	89.71 (3)	C4—C5—C6	116.23 (14)
Cl1—Cd—Cl2^i^	86.682 (15)	C4—C5—H5B	108.2
Cl2—Cd—Cl2^i^	174.893 (8)	C6—C5—H5B	108.2
Cl1^ii^—Cd—Cl2^i^	96.408 (14)	C4—C5—H5A	108.2
Cd—Cl1—Cd^i^	94.075 (14)	C6—C5—H5A	108.2
Cd—Cl2—Cd^ii^	92.210 (15)	H5B—C5—H5A	107.4
C2—N1—C1	105.45 (14)	N3—C6—C5	113.23 (13)
C2—N1—Cd^ii^	126.08 (10)	N3—C6—H6A	108.9
C1—N1—Cd^ii^	128.47 (11)	C5—C6—H6A	108.9
C9—N2—C8	105.57 (13)	N3—C6—H6B	108.9
C9—N2—Cd	128.50 (11)	C5—C6—H6B	108.9
C8—N2—Cd	125.78 (10)	H6A—C6—H6B	107.7
C9—N3—C7	107.04 (13)	C8—C7—N3	105.85 (14)
C9—N3—C6	127.11 (13)	C8—C7—H7A	127.1
C7—N3—C6	125.75 (14)	N3—C7—H7A	127.1
C2—N4—C3	106.95 (13)	C7—C8—N2	110.02 (15)
C2—N4—C4	126.73 (15)	C7—C8—H8A	125.0
C3—N4—C4	126.12 (14)	N2—C8—H8A	125.0
C3—C1—N1	109.92 (15)	N2—C9—N3	111.52 (14)
C3—C1—H1B	125.0	N2—C9—H9A	124.2
N1—C1—H1B	125.0	N3—C9—H9A	124.2
N1—C2—N4	111.63 (15)		
			
N2—Cd—Cl1—Cd^i^	88.81 (4)	C3—N4—C2—N1	−1.12 (19)
N1^i^—Cd—Cl1—Cd^i^	−84.57 (3)	C4—N4—C2—N1	−176.17 (14)
Cl2—Cd—Cl1—Cd^i^	−179.129 (15)	N1—C1—C3—N4	0.3 (2)
Cl2^i^—Cd—Cl1—Cd^i^	4.723 (13)	C2—N4—C3—C1	0.47 (18)
N2—Cd—Cl2—Cd^ii^	−81.21 (4)	C4—N4—C3—C1	175.56 (16)
N1^i^—Cd—Cl2—Cd^ii^	91.81 (3)	C2—N4—C4—C5	−105.45 (19)
Cl1—Cd—Cl2—Cd^ii^	−171.071 (13)	C3—N4—C4—C5	80.4 (2)
Cl1^ii^—Cd—Cl2—Cd^ii^	4.639 (13)	N4—C4—C5—C6	79.6 (2)
Cl1—Cd—N2—C9	36.26 (14)	C9—N3—C6—C5	114.19 (19)
Cl2—Cd—N2—C9	−53.70 (14)	C7—N3—C6—C5	−61.6 (2)
Cl1^ii^—Cd—N2—C9	−140.18 (14)	C4—C5—C6—N3	−63.3 (2)
Cl2^i^—Cd—N2—C9	122.94 (14)	C9—N3—C7—C8	−0.01 (19)
Cl1—Cd—N2—C8	−148.84 (13)	C6—N3—C7—C8	176.46 (15)
Cl2—Cd—N2—C8	121.21 (13)	N3—C7—C8—N2	−0.34 (19)
Cl1^ii^—Cd—N2—C8	34.72 (13)	C9—N2—C8—C7	0.57 (19)
Cl2^i^—Cd—N2—C8	−62.16 (13)	Cd—N2—C8—C7	−175.29 (11)
C2—N1—C1—C3	−0.95 (19)	C8—N2—C9—N3	−0.58 (19)
Cd^ii^—N1—C1—C3	179.52 (12)	Cd—N2—C9—N3	175.12 (11)
C1—N1—C2—N4	1.27 (18)	C7—N3—C9—N2	0.4 (2)
Cd^ii^—N1—C2—N4	−179.18 (10)	C6—N3—C9—N2	−176.03 (14)

Symmetry codes: (i) −*x*+3/2, −*y*+2, *z*−1/2; (ii) −*x*+3/2, −*y*+2, *z*+1/2.
